# Medical Student Mentorship for Undergraduate Students Underrepresented in Medicine Improves Confidence and Knowledge About Medical School Application

**DOI:** 10.7759/cureus.63366

**Published:** 2024-06-28

**Authors:** Hannah E Myers, Maya Muhanna, McKenzie E Maloney, Luke G Scanlan, Brittany Ange, Vanessa Spearman-McCarthy, Tania K Arora

**Affiliations:** 1 School of Medicine, Medical College of Georgia, Augusta University, Augusta, USA; 2 Surgery, Medical College of Georgia, Augusta University, Augusta, USA; 3 Medicine and Psychiatry, Medical College of Georgia, Augusta University, Augusta, USA; 4 Surgical Oncology, Medical College of Georgia, Augusta University, Augusta, USA

**Keywords:** peer-based research mentorship, peer mentorship, mcat, medical school, underrepresented in medicine, mentorship

## Abstract

Purpose

Applying to medical school is accompanied by significant barriers to prospective applicants. Students who are underrepresented in medicine (URiM) may face additional barriers. We created a mentorship program to pair pre-medical URiM students with medical student mentors. The purpose of this study was to determine if providing mentorship and resources to URiM pre-medical students increased their knowledge and confidence regarding the medical school application process.

Method

A survey was emailed to mentees of the program to assess their knowledge and confidence about the Medical College Admission Test (MCAT) and medical school application before and after receiving mentorship. Wilcoxon-Signed-Rank tests were used for data analysis.

Results

A total of 28 participants completed the pilot study of which 17 gave qualitative feedback. Students reported feeling significantly more knowledgeable and confident after six months of enrollment on seven (77.8%) of the survey items. Respondents agreed that mentorship was the most valuable aspect of the program, with 13 (76.5%) respondents qualitatively endorsing the positive impact mentorship imparted to them.

Conclusion

Having a medical student mentor helped URiM pre-medical students feel more knowledgeable and confident about the medical school application process. By providing URiM students with additional resources, the diversity of future classes of physicians may improve and better mirror the populations they will serve.

## Introduction

The role of a physician is one of the most demanding careers an individual can choose, and the application process to medical school is no exception. Students face abundant challenges when applying to medical school including cost, entrance exams, written statements, letters of recommendation, and a rising number of applicants post-COVID-19 competing for limited program spots [1,2]. In 2020, there was approximately a 41% acceptance rate for students applying to medical schools, meaning that over half (59%) of applicants did not matriculate [3]. This statistic underlies the reality of the competition inherent in applying to medical school, which only worsened in the context of COVID-19 as the number of applications increased by 15% from 2019 to 2021 [2]. While this increase is not fully understood, it potentially increased the level of scrutiny students are subjected to.

The barriers to entry into medical school are significant and may be compounded for some students who are underrepresented in medicine (URiM). URiM students may suffer from a lack of role models and/or faculty advocates as academic faculty also suffer from a lack of diversity [4]. Additionally, some URiM students do not have access to resources to obtain the assistance and information they need due to the effects of structural racism in the medical school application process [5,6]. URiM pre-medical students are less represented than statistically expected in medical school classes, and even with new programs organized to help solve this issue, there is still a well-reported gap of underrepresented groups in medical schools [6,7]. For example, in 2015, this gap was estimated to be a deficit of 113,758 Hispanic and 81,358 Black physicians [8]. However, mentorship programs, especially within the medical field, are well-established as successful interventions to overcoming barriers [9-12].

To help bridge the gap that URiM pre-medical students face, a mentorship program was established at Augusta University (AU). This program paired underrepresented pre-medical students with first-year medical students at the Medical College of Georgia (MCG). This program provided free resources (e.g., exam prep books, free tutoring), information in the form of webinars and handouts, and mentorship to improve the chances of these students to successfully matriculate into medical school. The objective of our study was to evaluate the effect of medical student-led mentorship on URiM pre-medical students, we surveyed students enrolled in the mentorship program about their feelings regarding their knowledge of and confidence in applying to medical school before and after six months of mentorship.

## Materials and methods

Program description

Advertisements for the program contained a QR code to a form in which students could indicate their desire to participate in the program. Medical students were recruited as mentors primarily through advertisements in class-wide group messaging apps, email, and by word of mouth; there were no prerequisites. Pre-medical undergraduate students were recruited as mentees primarily through emails sent out via pre-health advisors, the Honors College, URiM affinity clubs, and diversity offices. Mentees were enrolled if they considered themselves to be URiM. Our definition of URiM expanded upon the Association of American Medical College’s (AAMC) definition of racial, ethnic, and social populations that are underrepresented in medicine based on the proportion of their representation in the general public [13] to include anyone who self-identified as URiM.

Mentors were expected to meet with their mentees at least once per month to check in with them and offer advice. A script of pertinent topics was provided for the first meeting, and subsequent meeting discussions were left to the discretion of the mentee and mentor. Additionally, mentees were offered optional monthly online seminars regarding medical school admissions, free tutoring, study resources, and social events for mentees and mentors to meet in person.

From October 2021-September 2022, a total of 66 medical student mentors and 76 pre-medical URiM mentees were enrolled in the program. Due to the larger number of mentees than mentors, select mentors had multiple mentees while maintaining compliance with mentorship expectations.

Participants

To determine the impact of mentorship on URiM pre-medical student’s knowledge and confidence regarding the medical school application process, we surveyed pre-medical students who had been enrolled in the mentorship program for at minimum of six months. This study was approved by the AU Institutional Review Board (#1919112-4). The survey included a retrospective portion, asking participants to indicate their level of knowledge and confidence regarding the medical school application process before program enrollment, in addition to a portion, asking them to indicate their current level of knowledge and confidence after a minimum of six months of program enrollment. The data was collected via an electronic survey from September 2022 through January 2023.

All students enrolled in the program for at least six months received an e-mail link to the survey. Participants were not able to complete the survey without reading and accepting the initial consent statement. Eligibility criteria included participants who 1) were at least 18 years of age; 2) received at least six months of mentorship; 3) considered themselves as URiM; 4) were actively enrolled in the mentorship program as well as an active student at the undergraduate university. Participants who did not meet inclusion criteria were excluded.

Knowledge and confidence measures

Participants indicated their level of knowledge regarding the following aspects of the medical application process on a 5-point Likert scale ranging from 1-Not knowledgeable at all to 5-Extremely knowledgeable both before and after receiving mentorship: 1) structure of the Medical College Admission Test (MCAT); 2) overall timeline of applying to medical school; 3) requirements of the primary application; 4) requirements of the secondary applications.

Participants were asked to indicate their level of confidence on a 5-point Likert scale ranging from 1-Not confident at all to 5-Extremely confident for the following items both before and after receiving mentorship: 1) when you take the MCAT, you will not be distracted by the format/structure of the exam; 2) when you take the MCAT, you will be able to get the score you want, 3) will be able to successfully submit your application to medical school in a timely manner; 4) will be able to successfully write a well-composed and representative personal statement for medical school; 5) will be able to successfully write about your interests, experience, and extracurricular activities for your medical school application.

Qualitative survey

At the end of the survey, participants were allowed to write in what they felt was the most valuable aspect of the program and a potential area for growth. One author (H.M.) read qualitative responses in their entirety and identified potential, underlying themes in the content. To be designated a theme, there was one criterion: to hold a minimum of three unique responses across all responses pertaining to the theme. Theme frequency was reported.

Data analysis

Incomplete responses were not included in the statistical analysis. All statistical analyses were performed using SAS 9.4 (SAS Institute Inc., Cary, NC, USA). The statistical significance was assessed using an alpha level of 0.05. Descriptive statistics (means, standard deviations, frequencies, and percentages) were calculated for all survey questions. Likert scale responses were converted to ordinal data (Extremely knowledgeable = 5 to not knowledgeable at all = 1 and Extremely confident = 5 to Not confident at all = 1) for means and standard deviations. Due to the non-normal nature of the data, Wilcoxon-Signed-Rank tests were calculated to determine if students reported a difference in knowledge or confidence pre- (“were” items) and post- (“are” items) mentorship intervention.

## Results

Fifty-four total students began the electronic survey. Those that were excluded from the analysis included individuals who did not answer any survey items or answered only demographic items (N=24), and those who responded to the post (“are”) questions but not the pre (“were”) questions (N=2). After exclusion, there were 28 participants in the final dataset, yielding a response rate of 36.8%. Table 1 provides descriptive statistics of the demographic information and survey items. The majority of the included students were 18-20 years old (N=21, 75%), first- or third-year students (N=16, 57%), female (N=21, 75%), and Asian (N=10, 35.7%).

**Table 1:** Descriptive statistics of survey participants.

Survey Question	Level	N (%)
Do you consider yourself underrepresented in medicine?	Yes	28 (100%)
What is your age?	18	6 (21.43%)
	19	6 (21.43%)
	20	9 (32.14%)
	21	2 (7.14%)
	>21	5 (17.86%)
What year are you in college?	First year	8 (28.57%)
	Second year	4 (14.29%)
	Third year	8 (28.57%)
	Fourth year	2 (7.14%)
	Fifth year	2 (7.14%)
	Graduated	4 (14.29%)
Please specify your gender.	Female	21 (75.00%)
	Male	7 (25.00%)
	Non-Binary/ Other	0 (0%)
	Prefer not to say	0 (0%)
Please specify your ethnicity.	Asian	10 (35.71%)
	Black or African American	8 (28.57%)
	Hispanic	2 (7.14%)
	Other	1 (3.57%)
	White	7 (25.00%)

Table 2 gives the results of the Wilcoxon-Signed-Rank tests calculated to determine if post-responses differed from pre-responses. Students reported statistically significantly more knowledge or confidence post-mentorship on all survey items except two (N=7, 77.8%).

**Table 2:** Wilcoxon-signed-rank test results to determine if knowledge and confidence differed from before enrolling in the mentorship program to at least six months after enrollment. MCAT: Medical College Admission Test

	Mean (SD) Post	Mean (SD) Pre	Mean (SD) Post-Pre	p-value
How knowledgeable are/were you about the structure of the MCAT?	2.96 (1.26)	1.96 (1.14)	1.00 (1.31)	0.0003
How confident are you that, when you take the MCAT, you will not be distracted by the format/structure of the exam	3.07 (1.15)	2.21 (1.13)	0.86 (1.15)	0.0003
How confident are you that when you take the MCAT, you will be able to get the score you want?	2.54 (1.37)	2.07 (1.33)	0.46 (1.20)	0.0713
How knowledgeable are you about the overall timeline of applying to medical school?	2.75 (1.24)	2.00 (1.12)	0.75 (1.14)	0.0014
How confident are you that you will be able to successfully submit your application to medical school in a timely manner?	2.96 (1.17)	2.18 (1.12)	0.79 (0.88)	0.0001
How knowledgeable are you about the requirements of the primary application for medical school?	2.64 (1.16)	1.11 (1.20)	0.54 (0.84)	0.0041
How knowledgeable are you about the requirements of the secondary applications for medical school?	2.07 (1.21)	1.79 (1.07)	0.29 (0.85)	0.1377
How confident are you that you can successfully write a well-composed and representative personal statement for medical school?	2.75 (1.17)	2.04 (1.17)	0.71 (1.05)	0.0007
How confident are you that you will be able to successfully write about your interests, experience, and extracurricular activities for your medical school application?	2.96 (1.20)	2.11 (1.10)	0.86 (0.80)	0.0001

Themes from qualitative survey questions were analyzed, and the findings common to multiple respondents were described in Table 3. Seventeen respondents defined the most valuable part of the program, and 14 respondents defined areas of improvement for the program. Respondents agreed that mentorship was the most valuable aspect of the program, with 13 (76.5%) respondents endorsing the positive impact mentorship imparted to them. Participants also described their appreciation for additional resources, such as MCAT books, a free MSAR subscription, and tutoring, in their responses, with 8 (47.1%) respondents mentioning their appreciation for these resources. Finally, regarding potential areas for growth in MMOMP, mentees supported additional social events within the group with 4 (28.6%) respondents requesting additional social events in their feedback.

**Table 3:** Qualitative themes from survey responses. ^a^ There were 17 respondents for question 35. ^b^ There were 14 respondents for survey question 37. MMOMP: MCG MCAT Outreach and Mentorship Program; MCAT: Medical College Admission Test; MCG: Medical College of Georgia.

Theme	Quote(s)	Number in Agreement
Value of Mentorship	My mentor’s enthusiasm to help me. He helps me when I’m going through a lot of doubt and always encourages me. I honestly felt like I’ve made a genuine friendship.	76.5% (13/17) ^a^
	MMOMP has allowed me to have a mentor that guides me through tough decisions and allows me to prioritize which opportunities are specifically important for me.	-
	Having a mentor to share my fears with and who holds me accountable for doing what needs to be done.	-
Resources for Students	I also found it helpful to receive the free MCAT books.	47.1% (8/17) ^a^
	MMOMP provides its mentees with opportunities for volunteering, shadowing, and having workshops to guide you… in preparing for medical school applications.	-
Desire for Additional Social Events	I would like to have more in-person group meetings to better form the community.	28.6% (4/14) ^b^
	More bonding events overall.	-
	I think a small group social would be nice.	-

## Discussion

In light of the COVID-19 pandemic, the AAMC made several changes to the medical school application process, including increased Fee Assistance Programs (FAP), reduced length of the MCAT, and virtual interviews. As a result, there was approximately a 21% increase in first-time applicants resulting in the most diverse group of applicants, with the majority of applicants identifying as non-White [14, 15]. However, significant barriers for URiM students still exist as these students are more likely to attend schools without adequate resources to prepare them for the medical school application process, including tutoring, counseling, mentoring, and exposure to healthcare professions, leaving them less competitive during the application process [16]. Further, URiM students may be deterred from pursuing a career in medicine for several reasons, including a lack of mentorship [14].

Overall, our results showed that near-peer mentorship increased both URiM pre-medical students’ knowledge and confidence about the medical school application process. Importantly, the mentorship program successfully increased students’ knowledge of the structure of the entrance exam, the overall application timeline, and the components of the application, filling a critical gap for URiM students. We hypothesize that this knowledge and guidance from mentors will allow these students adequate time to prepare for the entrance exam and the application as well as engage in activities that will strengthen their application in the interim. Because, despite many medical schools implementing a holistic review of applicants, the proportion of URiM students accepted remains less than the proportion of these same minoritized groups in the general population [16,17], therefore, URiM applicants may particularly benefit from enhancing their resume.

Further, this mentorship program went beyond near-peer mentorship and included tutoring, free MCAT prep book sets, and webinars hosted by medical students. While many students indicated that this was an important aspect of the mentorship program, we found no significant difference in respondents’ confidence in obtaining their desired MCAT score despite having these resources available. These results may be explained in part by the diversity in age, current year in undergraduate education, and individual career timelines of the participants. Confidence in achieving a desired entrance exam score may pertain more to students actively preparing for the MCAT. Therefore, while all students were given a baseline knowledge of the medical school application and MCAT in their first session with their mentor, some students may have focused on other challenges of the application process or their undergraduate career. Due to the multitude of hurdles students must manage to gain acceptance into medical school, it is possible students simply did not focus on this particular aspect; thus, our results may underestimate the impact of mentorship on this dimension of the application.

Per the qualitative feedback, mentees valued the mentorship aspect of the program the most. Moreover, the participants indicated that they formed genuine friendships with their mentors and relied on them for difficult decisions and situations. For example, one mentee shared that the program "has allowed me to have a mentor that guides me through tough decisions and allows me to prioritize which opportunities are specifically important for me." Mentorship has been shown to have significant psychosocial and career benefits for mentees [18]. Similar to previous studies [18], we found that mentorship provided more than career guidance by providing friendship and emotional support, which may become especially important throughout the long and stressful process of applying to medical school. Unfortunately, URiM students have fewer mentors than those groups that are not underrepresented [18], highlighting the need for formalized mentorship programs for these populations.

We recognize that this work represents an approach to increasing opportunity and education in pathway programming that was led by students without funding and limited resources and represents an important example that is easily scaled across any medical institution and is an important model for near-peer mentoring. Through the implementation of student-run mentorship programs, other institutions could help increase the confidence and knowledge that URiM students have when applying to medical school. Increasing the proportion of URiM students in medical schools has several downstream effects, including increasing racial and ethnic competency among medical students and improving outcomes for minoritized patients. For example, in a study of US allopathic graduating medical students, white students from schools in the highest diversity quintile felt more prepared to care for minoritized populations and had stronger attitudes endorsing equitable access to healthcare compared to students from the lowest diversity quintile schools [19]. Additionally, there is increasing evidence that minoritized patients directly benefit from racially and ethnically similar providers through improved communication, satisfaction, and treatment adherence [19]. By providing a free, accessible mentorship program to URiM pre-medical students, the diversity of future classes of physicians may improve and better mirror the populations they will serve.

A major limitation of this study was a lack of longitudinal follow-up. The survey included a retrospective portion, asking participants to recall their feelings of confidence and level of knowledge regarding the medical school application process before receiving mentorship a minimum of six months in the past. Therefore, our results were skewed by some level of recall bias. A small sample size also limited this pilot study, potentially underreporting the effects of the mentorship program. The low response rate was combated with several reminder emails to complete the survey. Further, because this was a small pilot study at a public school in the Southern United States, these results may not be generalizable to other peer mentorship programs in other settings. Additionally, we did not evaluate the matriculation rates of students. Knowledge of and confidence in applying to medical school are not synonymous with matriculation. Therefore, future studies should explore the short-term outcomes through prospective study designs and the long-term outcomes of formalized near-peer mentorship programs in terms of matriculation with larger sample sizes.

## Conclusions

In conclusion, URiM students are an essential part of the healthcare system that currently lacks the diversity of the population it serves. The barriers to applying to medical school are increased for these groups, and steps should be taken to decrease the disadvantages experienced by students in these populations to allow for better representation in medical school classes. Mentorship programs, such as the one described, are vital in bridging gaps for these students, providing them with the resources, knowledge, and confidence to level the application process.
